# What are the pathways between poverty and malaria in sub-Saharan Africa? A systematic review of mediation studies

**DOI:** 10.1186/s40249-023-01110-2

**Published:** 2023-06-08

**Authors:** Solomon T. Wafula, Theresa Habermann, Mara Anna Franke, Jürgen May, Dewi Ismajani Puradiredja, Eva Lorenz, Johanna Brinkel

**Affiliations:** 1 Department of Infectious Disease Epidemiology, Bernhard Nocht Institute for Tropical Medicine, Hamburg, Germany; 2 Department of Disease Control and Environmental Health, School of Public Health, Makerere University, Kampala, Uganda; 3 Charité Global Health, Charité Universitätsmedizin Berlin, Berlin, Germany; 4 German Center for Infection Research (DZIF), Partner Site Hamburg-Borstel-Lubeck-Riems, Hamburg, Germany; 5 Department of Tropical Medicine, University Medical Center Hamburg-Eppendorf (UKE), Hamburg, Germany; 6 Institute of Medical Biostatistics, Epidemiology and Informatics, University Centre of the Johannes Gutenberg University, Mainz, Germany

**Keywords:** Malaria, Mediation, Socioeconomic disparity, Sub-Saharan Africa

## Abstract

**Background:**

Malaria remains a major burden in sub-Saharan Africa (SSA). While an association between poverty and malaria has been demonstrated, a clearer understanding of explicit mechanisms through which socioeconomic position (SEP) influences malaria risk is needed to guide the design of more comprehensive interventions for malaria risk mitigation. This systematic review provides an overview of the current evidence on the mediators of socioeconomic disparities in malaria in SSA.

**Methods:**

We searched PubMed and Web of Science for randomised controlled trials, cohort, case-control and cross-sectional studies published in English between January 1, 2000 to May 31, 2022. Further studies were identified following reviews of reference lists of the studies included. We included studies that either (1) conducted a formal mediation analysis of risk factors on the causal pathway between SEP and malaria infections or (2) adjusted for these potential mediators as confounders on the association between SEP and malaria using standard regression models. At least two independent reviewers appraised the studies, conducted data extraction, and assessed risk of bias. A systematic overview is presented for the included studies.

**Results:**

We identified 41 articles from 20 countries in SSA for inclusion in the final review. Of these, 30 studies used cross-sectional design, and 26 found socioeconomic inequalities in malaria risk. Three formal mediation analyses showed limited evidence of mediation of food security, housing quality, and previous antimalarial use. Housing, education, insecticide-treated nets, and nutrition were highlighted in the remaining studies as being protective against malaria independent of SEP, suggesting potential for mediation. However, methodological limitations included the use of cross-sectional data, insufficient confounder adjustment, heterogeneity in measuring both SEP and malaria, and generally low or moderate-quality studies. No studies considered exposure mediator interactions or considered identifiability assumptions.

**Conclusions:**

Few studies have conducted formal mediation analyses to elucidate pathways between SEP and malaria. Findings indicate that food security and housing could be more feasible (structural) intervention targets. Further research using well-designed longitudinal studies and improved analysis would illuminate the current sparse evidence into the pathways between SEP and malaria and adduce evidence for more potential targets for effective intervention.

**Graphical Abstract:**

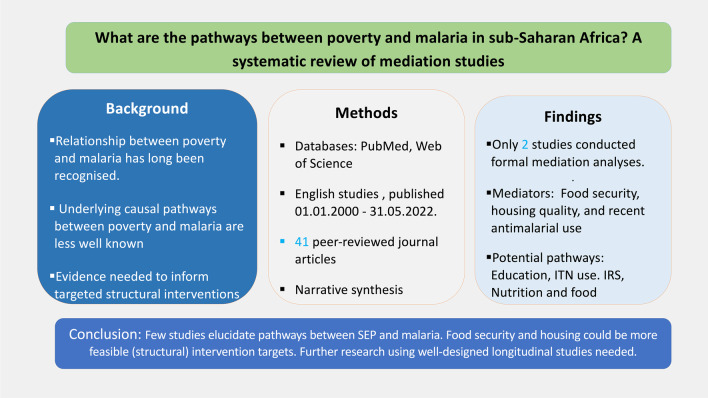

**Supplementary Information:**

The online version contains supplementary material available at 10.1186/s40249-023-01110-2.

## Background

Malaria is considered a disease of poverty [1]. Approximately 90% of all malaria-related morbidity and mortality occur in the world’s poorest regions, such as sub-Saharan Africa (SSA) [2]. Evidence of socioeconomic inequalities in the malaria burden has been consistently documented [1, 3]. Recent systematic reviews show that greater household wealth is associated with reductions in malaria [4–6]. For instance, evidence indicates that the risk of malaria is halved in children from the least poor households compared with those from the poorest households [4, 7]. Previous studies have used different proxies such as education, urbanicity, occupation, housing, and income to define socioeconomic position (SEP) [4, 6]. These proxies are either difficult to measure, have untestable assumptions about the link between indicators and poverty or sometimes comparisons across contexts/settings are not always valid. While household consumption is a better measure than income because it is less affected by inflation, measuring it is time-consuming and subject to bias [8]. A recently validated methodological approach that employs wealth indices derived from household assets, housing and living conditions is rarely used [9].

**Fig. 1:**
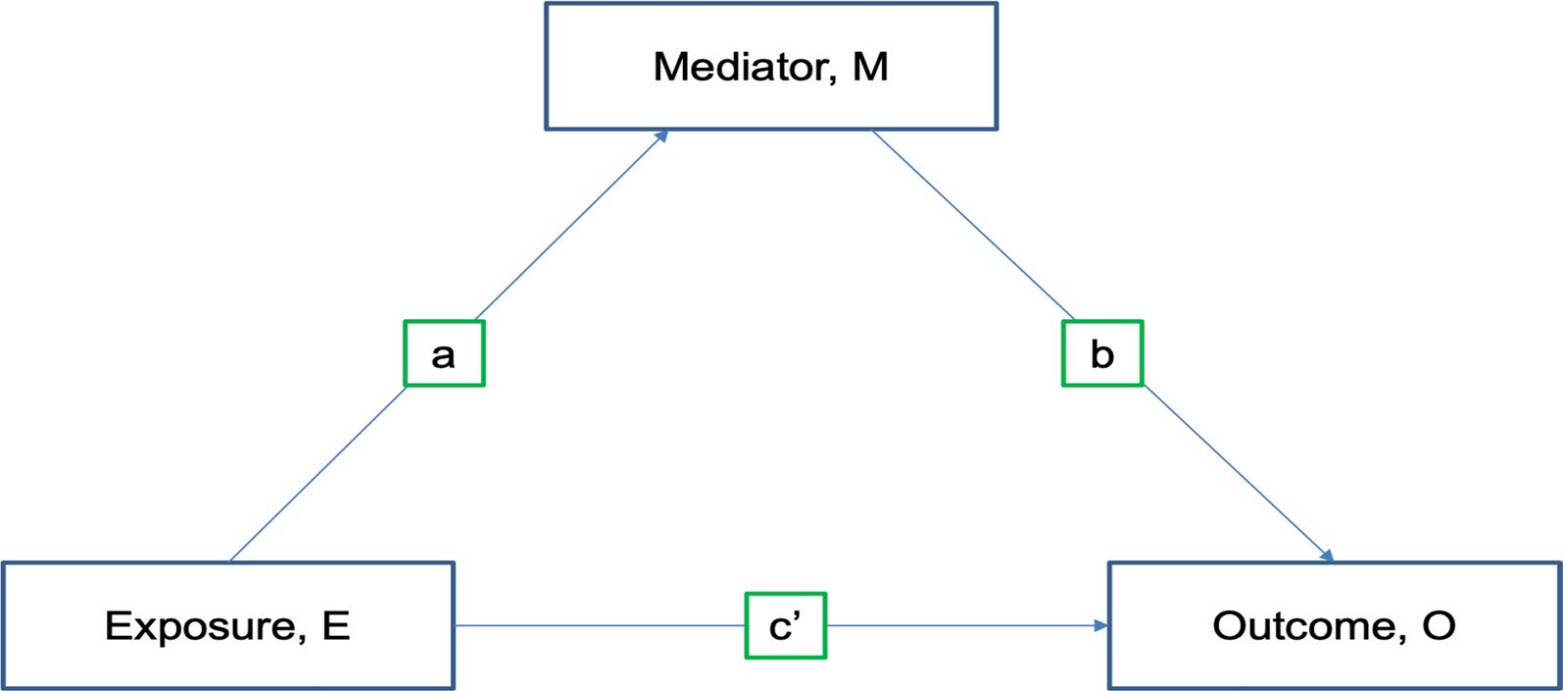
Relationship between exposure E, mediator M and outcome O

However, the impact of improved SEP on malaria may be largely indirect [1]. Indeed, studies show that socioeconomic disparities in malaria may be partly explained by factors on the causal pathway, such as improved housing, education, nutrition, food security, and use of insecticide-treated nets (ITN) [1, 10, 11]. If a causal relationship exists between SEP and malaria, then mediating pathways between SEP and malaria may be viable targets for interventions to reduce malaria incidence. Mediation analysis helps to understand whether and to what extent a third (intermediate) variable explains an exposure’s effect by partitioning the total effect of exposure into direct and indirect effects [12, 13]. The mediation analysis is depicted in Fig. 1 where a = coefficient of the path from exposure (E) to mediator (M), b = coefficient of the path from M to outcome (O) and c′ = coefficient of the path from E to O. The path c′ is the direct effect of E on O while the indirect effect of E is through a and b [14].

The difference method and the product of coefficients method are two conventional approaches biomedical researchers have used to conduct mediation analysis, but they both have drawbacks. The recent developments in the causal inference literature have made it possible to conduct mediation analysis with exposure-mediator interactions, multiple mediators, and counterfactual outcome perspectives [14]. In light of these methodological developments, reviewing the current evidence of the mediators between SEP and malaria provides helpful information to guide future analyses.

Although extant evidence supports the association between SEP and malaria, there is a lack of detailed studies to elucidate the underlying mechanisms behind this association and assess the evidence in light of the recent developments in mediation analysis. To our knowledge, there is no systematic review of studies that apply mediation analyses to investigate the underlying mechanisms between SEP and malaria. There is also a lack of synthesis of studies in which potential mediators have been adjusted for in the analyses. Therefore, this systematic review aims to comprehensively identify, summarize and critically appraise the existing evidence regarding variables that potentially mediate the relationship between SEP and malaria in SSA. Highlights of the review have been provided (Additional file 1: Table S1).

## Methods

### Search strategy and selection process

This review was conducted and reported following the Preferred Reporting Items for Systematic Reviews and Meta-Analysis protocols (PRISMA-P) 2020 statement [15] and registered in PROSPERO in March 2022 (Registration ID: CRD42022312359). We searched PubMed and Web of Science (WoS) for studies published in the English language for the period between January 1, 2000 and June 30, 2020 and updated in May 2022. A search strategy was developed to identify studies reporting on mediators of socioeconomic inequalities in malaria in SSA. A list of search terms and a detailed search strategy are provided (Additional file 1: Table S2).

### Inclusion and exclusion criteria

We included all studies that quantitatively assessed mediators of SEP (derived from asset ownership and water and sanitation status) and malaria. We also included studies that reported associations between SEP and laboratory-confirmed malaria and simultaneously included potential mediators as covariates. The study population was not restricted to any age or gender, provided the studies were conducted in SSA [16]. We considered peer-reviewed articles published between January 1, 2000, and May 31, 2022, as eligible for inclusion. Studies that only considered one dimension of SEP, such as income, education, housing, occupation, and those using self-reported malaria or fever as a proxy for malaria, were excluded from this review. We excluded editorials, commentaries, conference abstracts, protocols, case reports and narrative reviews.

### Identification of studies and data extraction

The screening was done at different stages. First, the authors (MAF and THH) screened all titles and abstracts of retrieved articles. We evaluated full texts when the abstract was deemed insufficient to draw conclusions. Full texts were then screened by three independent reviewers (STW, MAF, and THH) who extracted all relevant information into a standardized Excel spreadsheet. We also searched existing systematic reviews and reference lists of identified studies in addition to the electronic search. For excluded studies, reasons for exclusion were recorded. In case of discordance, the question of the inclusion of articles was resolved by a discussion with a reviewer panel (EL, JB, DIP). The comprehensive search results were merged, and duplicates were verified and removed.

Two independent reviewers (STW and THH) extracted relevant data from each paper using prepared data extraction forms to summarise evidence after the full text screening. We collected information on the first author's name, country of origin, study designs, study settings, sample size and participant characteristics (age, gender, domain), sampling methods, indicators of malaria and SEP, effect estimates (i.e., odds ratios, risk ratios, highest posterior densities), analysis methods (including mediation), covariates, and limitations. For studies that performed formal mediation analyses, we captured data on the percentage of the total effect that was mediated in each pathway.

### Study quality assessment

Two authors (SWT and MAF) undertook quality assessment using an adapted version of the Effective Public Health Practice Project Tool (EPHPP) [17] (Additional file 1: Table S3). We evaluated the quality of individual studies based on participant selection, study design, control of confounding, measurement of outcome, assessment of the exposures, and withdrawals and drop-outs (for longitudinal studies). We rated each item as weak, moderate, or strong according to the quality assessment criteria and determined an overall global rating for the included studies. We categorized studies into strong, moderate, and weak based on the criteria. Studies with no weak component ratings were assigned as “strong”. Those with one weak component ratings were assigned “moderate”, while those two or more weak component ratings received “weak” quality ratings. We resolved any discrepancies through discussion. Details of the ratings are available (Additional file 1: Table S4).

### Data synthesis strategy

Due to significant heterogeneity in the studies in terms of study designs, study populations, and settings, a comprehensive narrative synthesis was performed to answer the review’s objective. Study findings have been presented in tabular form, highlighting country, year of study, study population, context, mediators considered, and outcome measurement, among others.

### Patient and public involvement

No patients were involved in the conceptualisation or conduct of this study due to the nature of the study as a systematic review.

## Results

### Search results and eligible studies

**Fig. 2:**
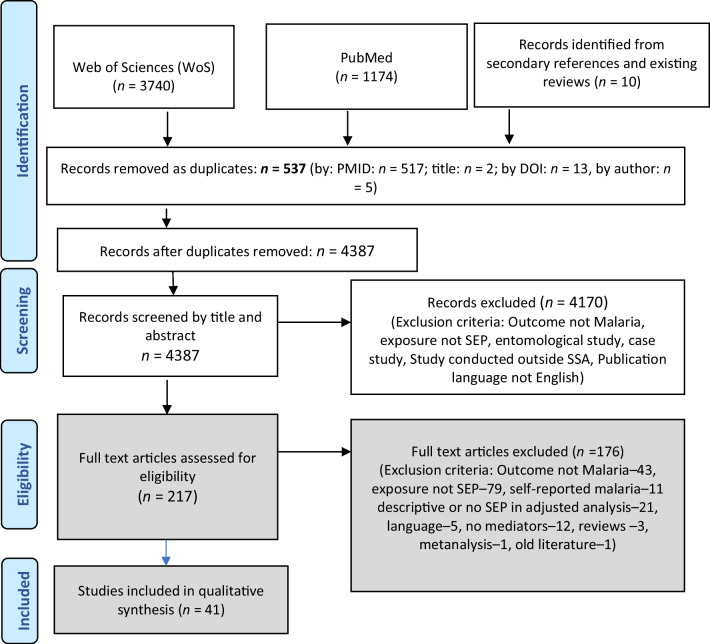
PRISMA flow diagram for study screening and selection process. *DOI* digital object identifier, *PMID* PubMed identifier*, SEP* socioeconomic position, *WOS* web of science

A total of 4914 articles were obtained after searching literature from the two databases. An extra 10 articles were identified by reviewing references of included studies [18–23] and relevant recent systematic reviews [24–27]. Of the 4924 articles, 537 were found to be duplicates. After screening the titles and abstracts, 217 were retained and determined as eligible for full text review resulting into 176 articles being excluded and leaving 41 articles. A flow chart including details of the article screening process is shown (Fig. 2).

### Characteristics of included studies

Forty-one (41) studies were conducted in 20 countries in SSA. The review had eight studies conducted in Tanzania [21, 23, 28–33], and Malawi [23, 34–40] and seven studies from Uganda [1, 22, 23, 25, 41–43], four in Ethiopia [18, 19, 44, 45], three in Kenya [46–48], two in Ghana [27, 49], the Gambia [27, 49], and Burkina Faso [49, 50], Democratic Republic of Congo (DRC) [51, 52], Nigeria [23, 53], Mali [49, 54], Mozambique [55, 56], while Cameroon [57], Equatorial Guinea [24], Angola [23], Liberia [23], Rwanda [23], Senegal [23], Madagascar [23] and South Africa [20] all had one study.

Of the 41 studies, 30 used a cross-sectional design [19, 23–25, 27, 28, 30–40, 42, 45–47, 49–53, 55–58], seven used a cohort design [1, 18, 22, 26, 41, 43, 54], two were case–control studies [20, 48], and two investigations were embedded trials [29, 44]. All studies used objective diagnostic malaria tests, and most studies (26) had children as their population, eight had the general population, six considered both children and their caretakers, and two considered adult women. The characteristics of included studies are included in Table 2.

### Measures of SEP

All studies used wealth indices constructed from household assets and other variables to measure SEP per our definition of SEP. Unlike most studies which considered reported SEP in quintiles, quartiles and tertiles for wealth index, some studies also considered SEP as a continuous variable [18, 19, 25, 30, 31, 39, 46, 47, 51, 57], wealth percentiles [23, 48], or as a dichotomous variable [50].

### Measures of outcome: malaria

Malaria was assessed as the prevalence or incidence of *Plasmodium* infection**.** Twenty-six studies used microscopy to test for malaria, 12 used rapid diagnostic tests (RDTs), 2 used polymerase chain reaction (PCR) and 1 performed histopathology for diagnosis of placental malaria (Table 1).

**Table 1:** The characteristics of included studies in the present review

Author year; country	Study design	Study population	Sample size, *n*	Indicator of SEP	Outcome measurement
Siri 2010; Kenya	Case-control	Cases were children less than 10 years with a hemoglobin level < 8 g/dL and parasite density ≥ 10,000/μl and are normal residents	906	Wealth Index (continuous)	Malaria examined by microscopy
Coleman et al. 2010; South Africa	Case-control	All Household members (household considered instead of individuals)	212	Wealth Index (quartile)	Malaria examined by microscopy
Loha et al. 2012; Ethiopia	Cohort	All residents in the Kebele were taken as study subjects. Every household was visited weekly, looking for febrile cases	8121	Wealth Index (continuous)	Malaria RDT
Clark et al. 2008; Uganda	Cohort	Children aged 1–10 years presenting with fever episode	601	Wealth Index (quartile)	Malaria examined by microscopy
Snyman et al. 2015; Uganda	Cohort	HIV-exposed and unexposed children of age 4–6 months	515	Wealth Index (tertiles)	Malaria examined by microscopy
Tusting et al. 2016; Uganda	Cohort	All children aged 6 months to 10 years and their primary caregivers	333	Wealth Index (tertile)	Malaria examined by microscopy
Wanzirah et al. 2015; Uganda	Cohort	All children aged 6 months to 10 years	878	Wealth Index (tertile)	Malaria examined by microscopy
Asante et al. 2013; Ghana	Cohort	Infants of mothers enrolled during pregnancy	1855	Wealth Index (quintile)	Malaria RDT and Malaria examined by microscopy
Vincenz et al. 2022; Mali	Cohort	Mothers who were participants in a cohort study	249 mothers	Wealth z scores	Placental malaria (histology)
Haji et al. 2016; Ethiopia	Cross-sectional	All children under 16 years with symptoms consistent with malaria	830	Wealth Index (tertiles)	Malaria examined by microscopy
Kabaghe et al. 2017; Malawi	Cross-sectional	Children 6–59 months enrolled	1016	Wealth index (continuous)	Malaria RDT
Mathanga et al. 2015; Malawi	Cross-sectional	Pupils enrolled in classes 1–8 of participating schools	2667	Wealth Index (quintiles)	Malaria examined by microscopy
Sakwe et al. 2019; Cameroon	Cross-sectional	Children aged 6 months to 10 years and of both sexes, after full informed consent	362	Development Index)—(continuous)	Malaria examined by microscopy
Skarbinski et al. 2011; Malawi	Cross-sectional	All household members were asked to participate	6581	Wealth Index (quintiles)	Malaria examined by microscopy
Skarbinski et al. 2012; Malawi	Cross-sectional	All household members were asked to participate	884	Wealth Index (quintiles)	Malaria was examined by microscopy
Somi et al. 2007; Tanzania	Cross-sectional	Children and adults	7657	Wealth Index (continuous)	Malaria was examined by microscopy
Somi 2008; Tanzania	Cross-sectional	General population	2034	Wealth index (continuous)	Malaria was examined by microscopy
Ssempiira et al. 2017; Uganda	Cross-sectional	Women 15–49 years, Children less than 5 years	4591 children	Wealth Index (quintiles)	Malaria RDT
Temu et al. 2012; Mozambique	Cross-sectional	Children 1–15 years	8338 children	Wealth Index (quartiles)	Malaria RDT
Zoungrana et al. 2014; Burkina Faso	Cross-sectional	All children 0–5 years who had been diagnosed with clinical malaria or produced a paraclinical assessment	510	Wealth Index (dichotomous)	Malaria RDT or Malaria examined by microscopy
Graves et al. 2009; Ethiopia	Cross-sectional	Children and women	11,437	Wealth Index (continuous)	Malaria RDT
Mmbando et al. 2011; Tanzania	Cross-sectional	children 0–19 years	12,298	Wealth Index (tertile)	Malaria examined by microscopy
Siri, 2014; 9 SSA countries	Cross-sectional	Children	34,139	Wealth Index (continuous)	Malaria RDT and Malaria examined by microscopy
Custodio et al. 2009; Equatorial Guinea	Cross-sectional	Children 0–5 years old	552	Wealth Index (tertile)	Malaria examined by microscopy
de Beaudrap et al. 2011; Uganda	Cross-sectional	Children 0–5 years old	2847	Wealth Index (continuous)	Malaria RDT and Malaria examined by microscopy
Sonko et al. 2014; The Gambia	Cross-sectional	Children and the general population	6–59 months (*n* = 1248), 5 to 14 years (*n* = 1987), adults (*n* = 2306)	Wealth Index (quintile)	Malaria RDT and Malaria examined by microscopy
Chirombo et al. 2014; Malawi	Cross-sectional	Children under 5 years	2093	Wealth Index (quintiles)	Malaria RDT
de Glanville et al. 2019; Kenya	Cross-sectional	Individuals older or equal to 5 years	2113	Wealth Index (continuous)	Malaria examined by microscopy
Florey et al. 2012; Kenya	Cross-sectional	Children aged 8–17 and adults 18–86	223 children and 338 adults	Wealth index (continuous)	Polymerase chain reaction
Kahabuka et al. 2012; Tanzania	Cross-sectional	Caretakers who brought their sick children at Korogwe and Muheza district hospitals	296	Wealth Index (tertiles)	Malaria RDT
Ma et al. 2017; DRC	Cross-sectional	Healthy children aged 2 months to 5 years attending well-child and/or immunization visits	647	Wealth index (continuous)	Malaria RDT
West et al. 2013; Tanzania	Cross-sectional	Children 6–14 years	5152	Wealth index (cluster SEP) (tertiles)	Malaria RDT
Williams et al. 2016; Burkina Faso, The Gambia, Ghana and Mali	Cross-sectional	Women enrolled in a trial of intermittent screening and treatment of malaria in pregnancy (ISTP) versus IPTP	2526	Wealth Index (quintiles)	Malaria RDT and Polymerase Chain Reaction
Zgambo et al. 2017; Malawi	Cross-sectional	Children under the age of five	2012 Survey (*n* = 2173), 2014 Survey (*n* = 2029)	Wealth index (quintiles)	Malaria examined by microscopy
Mann et al. 2021; Nigeria	Cross-sectional	Children aged 6–59 months	12,996	Wealth index (quintile)	Malaria examined by microscopy
Emina et al. 2021; DRC	Cross-sectional	Children aged 6–59 months	8547	Wealth index (quintile)	Malaria examined by microscopy
Mwaiswelo et al. 2021; Tanzania	Cross-sectional	Children aged 3–59 months	2340	Wealth index (tertiles)	Malaria RDT
Mangani et al. 2022; Malawi	Cross-sectional	All individuals aged 6 months or older who slept in the house for at least 2 weeks of the previous month	5829	Wealth Index (quintiles)	Malaria RDT
Ejigu 2020; Mozambique	Cross-sectional	Children of age 6–59 months	4347	Wealth Index (quintiles)	Malaria RDT
Gari et al. 2016; Ethiopia	Trial	General population	5309	Wealth Index (tertiles)	Malaria RDT or Malaria examined by microscopy
Liu et al. 2014; Tanzania	Trial	Children (one per household) were followed from 2 to 24 months	435	Wealth Index (quintiles)	Malaria examined by microscopy

*DRC* Democratic Republic of Congo, *IPTP* intermittent preventive treatment in pregnancy, *RDT* rapid diagnostic test, *SEP* socioeconomic position

### Associations between SEP and malaria (regression and mediation analyses)

**Fig. 3:**
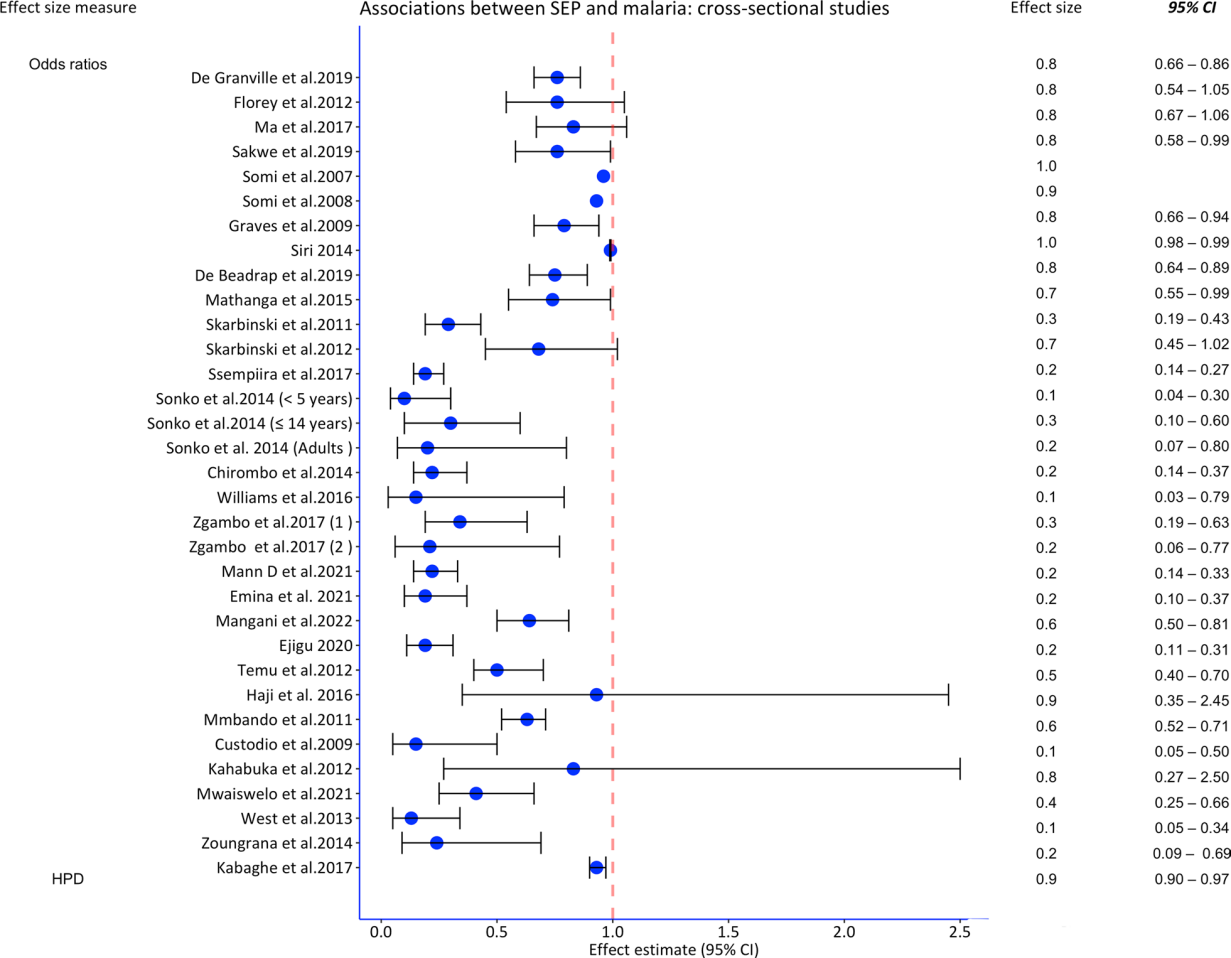
Forest plot of risk estimates from cross-sectional studies assessing the association between socioeconomic position and malaria in Sub-Saharan Africa. *CI* confidence interval, *HPD* highest posterior density, *SEP* socioeconomic position

**Fig. 4:**
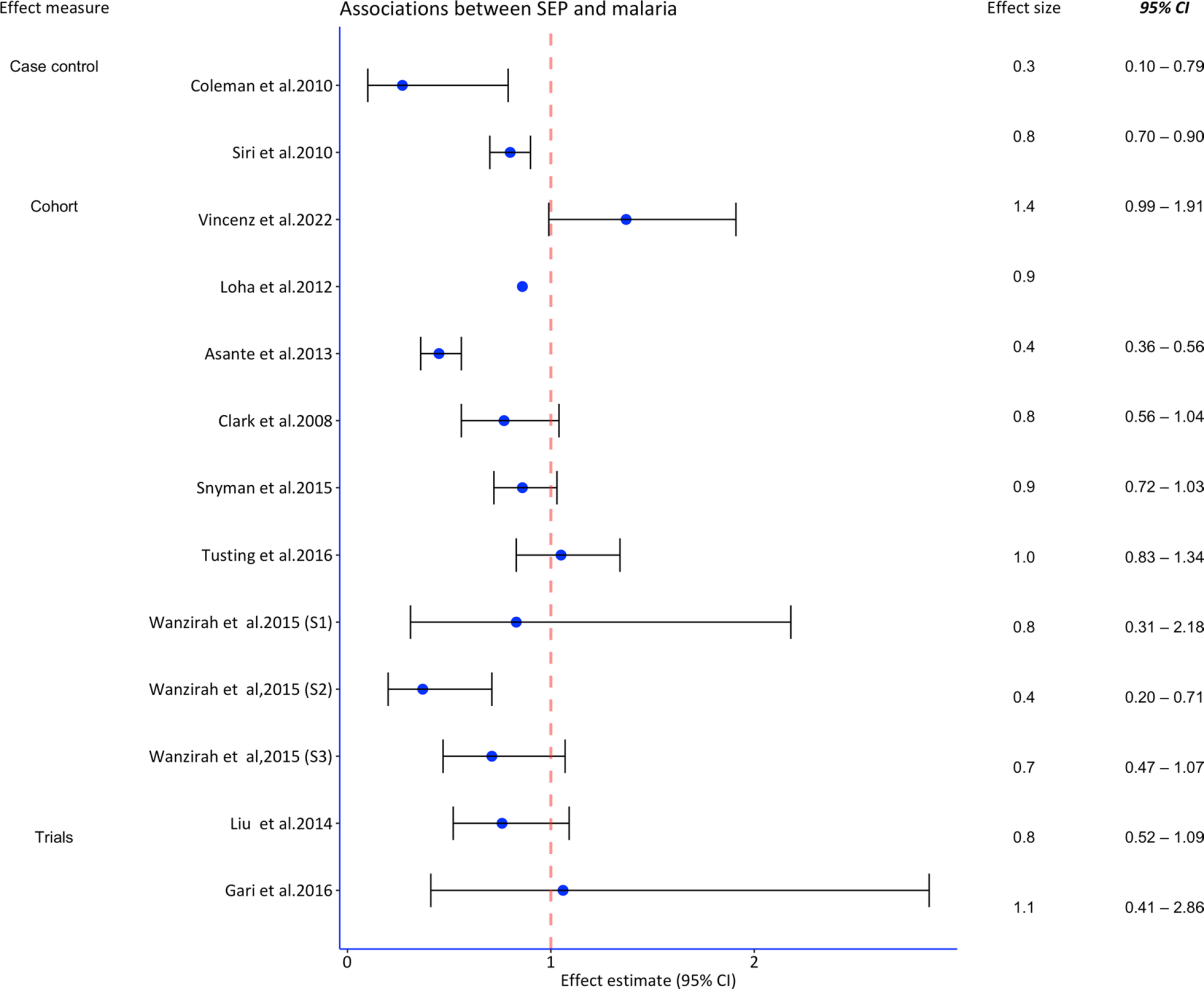
Forest plot of risk estimates from the cohort, case–control studies and trials for the association between socioeconomic position and malaria in Sub-Saharan Africa. *CI* confidence interval, *SEP* socioeconomic position

Most studies employed multivariable logistic regression, followed by Poisson regression. Two studies conducted three mediation analyses. Overall, 39 studies indicated a protective effect of higher SEP on malaria (point effect estimate). Of these, all cross-sectional studies, eight out of nine cohort/case-control studies, and one trial indicated a protective effect. The analyses performed, the effect estimates of SEP on malaria, the confounders adjusted form and quality ratings are shown in Table 2 and Figs. 3 and 4.

**Table 2:** Studies on socioeconomic position (SEP) and malaria with analysis approaches

Author year	Analyses performed	Risk estimate (95% *CI*) and result of mediation (if any)	Adjusted for confounders/mediators	Quality score
Siri 2010	Multivariable logistic regression	*OR.* wealth percentile [0.8 (0.7–0.9)]	ITN use, mosquito coils, age, location, gender of HH head	Strong
Coleman et al. 2010	Multivariable negative binomial regressions	*OR*. Reference (the 1st quartile) third [0.24 (0.09–0.65)], and fourth (least poor) [0.27 (0.10–0.79)]	Housing structure, closing windows on retiring	Strong
Loha et al. 2012	Poisson regression	(*Beta* coefficient = − 0.155, P-value = 0.043)	Age, gender, ITN use, and distance to the breeding place	Strong
Clark et al. 2008	Generalized estimating equations with control for repeated measures	*IRR*. Reference (SEP quartiles 1 and 2 combined). Third [0.83 (0.62–1.10)], fourth [0.77 (0.56–1.04)]	Age, gender, ITN use, distance to the swamp, household crowding	Strong
Snyman et al. 2015	Negative binomial regression models	*IRR.* Reference (lowest). Middle [0.91 (0.76–1.1)], highest [0.86 (0.72–1.03)]	Caregiver’s age, education, house construction, location, number of rooms	Moderate
Tusting et al. 2016	Multivariable logistic regression and causal steps approach-simulations and Bootstrapping	*IRR*. Reference (lowest), middle [1.12 (0.90–1.40)], highest [1.05 (0.83–1.34)] Housing type explained 24.9% of the SEP effect, and food security explained 18.6%	Age, gender, level of education, housing type^a^, food security^a^, distance to facility, household size	Strong
Wanzirah et al. 2015	Multivariable logistic regression and negative binomial regression	*OR*. Reference (1st tertile) Walukuba: 2nd tertile [0.82 (0.38–1.78)], 3rd tertile [0.83 (0.31–2.18)] Kihihi: 2nd tertile [0.54 (0.28–1.06)], 3rd tertile [0.37 (0.20–0.71)] Nagongera: 2nd tertile [0.72 (0.50–1.04)], 3rd tertile [0.71 (0.47–1.07)]	Age, gender, house type, floor material, roof material, eaves	Moderate
Asante et al. 2013	Cox proportional hazards regression	*HR*. Reference (least poor), less poor [1.54 (1.23–1.93)], poor [1.88 (1.50–2.35)], poorer [1.86 (1.50–2.31)], most poor [2.21 (1.77–2.76)]	Housing (thatched roof), location, distance to the health facility, ITN use	Strong
Haji et al. 2016	Multivariable logistic regression	*OR*. Reference (low), medium [1.51 (0.51–4. 45)], high [0.93 (0.35–2.45)]	Location, ITN use, age of the child, gender, sought advice before, knowledge of malaria	Moderate
Kabaghe et al. 2017	Modified Poisson regression	*HPD*. SEP [− 0.07 (− 0.11 to − 0.03)]	Age, ITN use, elevation and Normalized Difference Vegetation Index	Weak
Mathanga et al. 2015	Multivariable logistic regression	*OR*. Reference (poorest), poor [1.08 (0.82–1.42)], medium [1.30, 0.98–1.72)], less poor [1.26 (0.94–1.70)], least poor [0.74 (0.55–0.99)]	Age, gender, ITN use, reported fever, education status, household size, school feeding)	Moderate
Sakwe et al. 2019	Multivariable logistic regression	*OR*. Development Index [0.76 (0.58–0.99)]	Child age, gender, nutrition status, housing type, HH size, HH head education, and caregiver	Moderate
Skarbinski et al. 2011	Multivariable logistic regression	*OR*. Reference (least poor), 4th [2.10 (1.45–3.05)], 3rd [2.64 (1.80–3.87)], 2nd [2.84 (2.03–3.97)], 1st [3.46 (2.30–5.21)]	District, ITN use, IRN use	Weak
Skarbinski et al. 2012	Binomial regression modelling	*OR*. Reference (least poor), 4th [1.19 (0.71–2.00)], 3rd [1.72 (1.09–2.70)], 2nd [1.52 (1.01–2.29)], 1st [1.47 (0.98–2.20)]	IRS use, ITN use, wall material, roof material	Weak
Somi et al. 2007	Probit regression	Coefficients: SEP score based on PCA (numerical) = − 0.04 (*P*-value = 0.012)	ITN use, age, location, knowledge, eaves	Moderate
Somi et al. 2008	Probit regression	Coefficients: SEP score based on PCA (numerical) = − 0.074	Age, location, ITN use, HH size, eaves, knowledge	Moderate
Ssempiira et al. 2017	Bayesian geostatistical logistic regression	*OR*. Reference (poorest), richest [0.19 (0.14, 0.27)], richer [0.52 (0.42, 0.61)], medium [0.77 (0.85, 1.15)], poorer [0.86 (0.72, 1.04)]	Location, ITN use, IRS use, age, mothers’ education, land surface temperature	Moderate
Temu et al. 2012	Multivariable logistic regression	*OR.* Reference (poorest), 2nd quartile [0.9 (0.7–1.2)], 3rd quartile [0.9 (0.7–1.3)], and 4th quartile [0.5 (0.4–0.7)]	Age, year, ITN use, HH size, house construction, children with current fever	Weak
Zoungrana et al. 2014	Multivariable logistic regression	*OR.* Reference (high) Low SEP [4.11 (1.44, 11.75)]	Age, gender, marital status, education, knowledge, ethnicity, residence, distance, travel time, HH size, decision making	Strong
Graves et al. 2009	Multivariable logistic regression	*OR*. Asset index [0.79 (0.66—0.94)]	Age, gender, altitude, monthly rain, ITN use, IRS use in the last 12 months	Moderate
Mmbando et al. 2011	Muitivariate logistic regression Spatial analysis	*OR*. Reference (high), medium [1.6(1.3–1.9)], low [1.6 (1.4–1.91)]	Age, ITN use, ITN rate, housing, year, altitude	Moderate
Siri 2014	Multilevel logistic regression	*OR.* Wealth percentile [0.990 (0.987–0.992)]	Child age, mother’s age, ITN use, country, HH size, location, education, finished windows and ceilings	Moderate
Custodio et al. 2009	Multivariable logistic regression	*OR*. Reference (low), medium [0.97 (0.29–3.25)], high [0.15 (0.05–0.50)]	ITN use, antimalarials use in pregnancy, age, gender	Moderate
de Beaudrap et al. 2011	Multivariable logistic regression	*OR*. SEP score [0.75 (0.64–0.89)]	Child age, weight, housing score, ITN use, education and latitude	Moderate
Sonko et al. 2014	Multivariable logistic regression	For children 6–59 months, *OR*. Reference (poorest), 2nd [0.40 (0.20–0.70], 3rd [0.5 (0.30–0.90)], 4th [0.30 (0.10–0.60), 5th [0.10 (0.04–0.30)] Children 5–14 years 2nd [0.60 (0.30–1.20]), 3rd [0.70 (0.50–1.10]), 4th [0.20 (0.10–0.50)], 5th [0.30 (0.10–0.60)]. For the general population 2nd [0.80 (0.40–1.30)], 3rd [0.80 (0.50–1.20), 4th (0.40 (0.20–0.80), 5th [0.20 (0.07–0.80)]	Housing (wall type, roof type, floor type, window type,) age, gender	Moderate
Chirombo et al. 2014	Structured additive logistic regression (Bayesian approach)	*OR*. Reference (poorest), richest [0.22 (0.14–0.37)], richer (0.42 (0.28–0.64)], medium [0.66 (0.45–0.96)], poorer [1.10 (0.76–1.60)]	ITN use, region, age and location	Weak
de Glanville et al. 2019	Multivariable logistic regression Mediation analysis using a hierarchical approach	*OR*. SEP [0.76 (0.66–0.86)] Minimal mediation by antimalarial use	Gender, age, access to health care (antimalaria use^a^)	Moderate
Florey et al. 2012	GEE models with exchangeable correlation matrix and logistic distributions	*OR*. SEP [0.76 (0.54–1.05)]	Outdoor night activity	Weak
Kahabuka et al. 2012	Multivariable logistic regression	*OR*. Reference (high), middle [1.00 (0.40–2.80)], and low [1.20 (0.40–3.70)]	Education, parity, hospital travel time, use of near public health facility, source of the first treatment	Weak
Ma et al. 2017	Multivariable logistic regression	*OR*. SEP [1.20 (0.94–1.50)]	Study site, age, HH size, education, HIV Status, ITN use, phone ownership	Moderate
West et al. 2013	Multivariable logistic regression	*OR.* Reference (poorest), mild [0.69 (0.34–1.40)], least poor [0.13 (0.05–0.34)]	HH spaying, cluster ITN coverage, age	Moderate
Williams et al. 2016	Multivariable logistic regression	*OR.* Reference (wealthiest), wealthy [1.82 (0.68–4.83)], medium [0.96 (0.18–5.02)], poor [6.48 (1.68, 25.0), poorest [6.55 (1.27–33.70)]	Education, age, gestation age, gravidity, country	Weak
Zgambo et al. 2017	Multivariable logistic regression	*OR*. Reference (richest), 2012 survey: poorest [2.90 (1.60–5.30)], poorer [2.3 (1.10–4.60)], middle [2.50 (1.30–5.00)], richer [1.9 (1.10–3.60)] 2014 survey: poorest [4.7 (1.3–16.2)], poorer [2.9 (0.9–10.0)], middle [2.7 (0.7–10.2)], richer [1.9 (0.4–8.0)]	ITN use, ITN ownership, IRS, region, location, Gender, child age, altitude, and education of the mother	Moderate
Gari T et al. 2016	A multilevel mixed effects Poisson regression	*IRR.* Reference (rich). Poor [0.94 (0.35–2.45)], medium [0.70 (0.33–1.50)]	Age, gender, HH head education, ITN use	Strong
Liu et al. 2014	Multivariable negative binomial regressions	*IRR.* Reference (middle). Poorest [1.316 (0.915–1.891)], poorer [1.292 (0.876–1.905)], richer [1.090 (0.667–1.782)], richest [1.059 (0.533–2.103)]	Age, housing index, regular repellent use, ITN use, location, water source, electricity	Strong
Vincenz et al. 2022	GEE for binary logistic regression	*OR*. [1.37 (0.99–1.91)]	Maternal age, gravidity, IPTP use, education, season	Weak
Mann et al. 2021	Multivariable logistic regression	*OR.* Reference (richest), poorest [4.60 (3.05–6.96)], poorer [4.18 (2.81–6.19)], middle [3.27 (2.26–4.71)], richer [2.23 (1.55–3.21)]	Age, gender, residence, education, nutrition (stunting)	Moderate
Emina et al. 2021	Generalized estimating equations with control for repeated measures	*OR.* Reference (poorest), poorer [1.20 (0.95–1.52)], middle [1.00 (0.77–1.31)], richer [0.69 (0.50–0.96)], richest [0.19 (0.10–0.37)]	Gender, child’s age, mother education, ITN use, sex of HH head, type of residence, province of residence	Moderate
Mwaiswelo et al. 2021	Multivariable logistic regression	*OR.* Reference (low). Medium [0.54, 0.36–0.83)], upper [0.41(0.25–0.66)]	ITN ownership, HH size, education, district (location)	Weak
Mangani et al. 2022	Multilevel logistic regression	*OR.* Reference (poorest), poorer [0.80 (0.65–1.00)], middle [0.74 (0.56–0.99)], richer [0.80 (0.62–1.01)], richest [0.64 (0.50–0.81)]	HH wall, roof materials, education, ITN use, child’s age, gender, distance from the irrigation scheme	Moderate
Ejigu 2020	Geostatistical logistic model	*OR.* Reference (poorest), poorer [0.99 (0.80–1.25)], middle [0.67 (0.53–0.85)], richer [0.52 (0.40–0.69)], richest [0.19 (0.11–0.31)]	Province, mothers’ education, anemia, ITN use, age in months, ITN coverage, malaria incidence	Weak

*CI* confidence interval, *GEE* generalized estimating equations, *HH* household, *HPD* highest posterior density, *PCA* principal component analysis, *OR* odds ratios*, IRR* incidence rate ratios, *ITN* insecticide treated net, *IRS* indoor residual spraying

^a^Mediators assessed in formal analyses

### Mediators and mediation methods: association between SEP and malaria

Two studies (a cohort and cross-sectional) investigated putative mediating factors on the path from SEP to malaria. Three pathways were explored: access to healthcare (use of antimalarials) [46], housing quality, and food security [1]. Only one study had a theoretical framework for mediation analysis [1], and no study assessed for interactions or performed sensitivity analyses which are vital to verify findings. Approaches to mediation analysis that overcome the limitations of the difference and the product methods exist and should be used. Details of mediators and methods applied are reported below.

*Antimalarial use* In a cross-sectional study by de Glanville et al. [46], there was significantly increased use of antimalarials by individuals in wealthier households with relatively lower malaria risk. This study indicated minimal mediation by antimalarial use, although the proportion of the effect mediated was not shown. In the adjusted model, however, both socioeconomic index (*OR* = 0.76, 95% *CI* 0.66–0.87) and recent antimalarial use (*OR* = 0.67, 95% *CI* 0.46–0.96) were protective against malaria. In this study, the mediation method applied was a regression-based comparison of the models (assessing for attenuation of coefficients) upon inclusion of the mediators.

*Housing quality and food security* A prospective cohort study by Tusting et al. [1] explored the mediating role of housing and food security on the effect of SEP on malaria incidence in Uganda using the Monte Carlo simulation approach described by Imai [59]. Food security was defined as secure if a household had 3–7 days of meat in the menu compared to only 0–2 times. A theoretical framework guided the mediation analysis in Tusting’s study [1]. In their analysis, housing type explained 24.9% of the effect of SEP, while food security explained 18.6% of the total effect of SEP on malaria risk.

*Health expenditure* (used as a proxy for treatment-seeking behaviour) was also explored. Due to limited information (data available for only 57% of children), mediation analysis was not robust and reported no mediating role.

### Potential mediators in adjusted regression models involving SEP and malaria

Without performing a formal mediation analysis, several studies adjusted for factors that potentially mediate the SEP and malaria relationship. In these studies, SEP was either a covariate or primary independent variable (exposure). These factors satisfy at least two conditions [i.e. (1) have statistically significant relationship between exposure (X) and outcome (Y) in univariable regressions and (2) inclusion of the mediator variable (M) should reduce the direct effect of X on Y] to support mediation, according to Baron and Kenny [60] and warrant testing for the mediating role in future studies.

### Use of insecticide-treated mosquito nets

The review identified 26 studies which adjusted for ownership/use of ITNs in the association between SEP and malaria. Of these, 20 studies showed a protective effect on malaria among individuals that used mosquito nets compared to those that did not. Two studies [24, 42] found that ITNs were associated with a higher risk of malaria, while four studies [19, 44, 48, 51] found marginal or no association with malaria risk.

### Education attainment

Educational attainment refers to the highest level of education that a person has successfully completed. This includes no formal education, primary/elementary, secondary and tertiary or vocational. Eighteen studies that included educational level [categorized into three levels: none, primary, post primary (11 studies), four levels: none, primary, secondary, post primary (4 studies), two levels: none/primary, post primary (3 studies)], and SEP in models predicting malaria risk were identified. The effect of educational level on malaria risk was mixed with 12 studies [28, 33, 34, 37, 40–42, 51–53, 56, 57] indicating the protective effect of higher educational levels on malaria risk, and six studies [23, 25, 44, 49, 50, 54] showed no significant association with malaria.

### Housing quality

Housing was expressed based on quality of specific housing materials (good vs poor); walls (*n* = 5), roof (*n* = 5), windows (*n* = 2), floor (*n* = 1), or additive housing index (*n* = 3). Eleven studies included housing quality in the multivariable regression models involving the association between SEP and the prevalence or incidence of malaria. Although eight studies [20, 21, 25–27, 29, 41, 43] indicated a significant protective effect of housing on malaria risk after controlling for SEP, the remaining two studies showed no associations between housing and malaria [23, 57]. There were also mixed associations in the cross-sectional survey by Somi [30], where improved walls were associated with reduced odds of malaria, while improved roofs had no significant effect on the risk of malaria. Rather than looking at the independent effect of roofs, walls, windows, eaves, and ceilings on malaria, most studies assessed the combined effect of these structures. Using these structures, studies grouped houses as poor quality (thatched roofs, dirt floors, completely uncovered windows, no ceilings, rough or mud walls and open eaves) and high quality (iron/tile roofs, concrete/brick walls, closed eaves, screened windows and ceilings).

### Indoor residual spraying

The review identified five studies that adjusted for IRS in their multivariable models containing SEP. Of these, four studies showed that IRS was associated with a lower risk of malaria [32, 35, 36, 42]. Graves et al. [19] did not find a relationship between IRS and malaria risk whether assessed at a 6- or 12-month history of IRS.

### Health-seeking behaviour

Access to health care was defined in terms of choice of place for healthcare (facility vs home; far vs nearest facility), and duration between symptom onset and seeking of care. Three studies adjusted for self-reported health-seeking behaviours in their associations involving SEP and malaria prevalence [28, 45, 50]. Two studies showed that seeking facility care versus at-home care and receiving treatment for fever symptoms promptly reduced the risk of severe malaria [28, 50]. In contrast, a study in Ethiopia found no significant association between early treatment seeking and malaria risk [45]. It is important to note that these studies used different proxies of health-seeking behaviour, and outcomes were different for example in one study, the outcome was severe malaria and in another, it was uncomplicated malaria.

### Antimalarials, nutrition status and outside night activity

The review identified two studies that adjusted for antimalarials [24, 54], four that adjusted for nutritional status [24, 34, 54, 57], and one trial that controlled for repellent use [29]. All these studies highlighted the protective effect of antimalarial use, nutrition, and repellent use, respectively. In addition, one cross-sectional study showed no association between outdoor night activities and malaria [47].

### Quality of reviewed studies

Eligible studies were assessed for their methodological quality using the EPHPP tool. Eight (8) studies were rated as having high quality (strong), and 11 studies were rated as having low quality, with the majority of the included studies having a moderate methodological quality (*n* = 22). Eight of the included studies deemed confounding bias to be a serious threat. These studies only controlled for less than 60% of the significant confounders in the association between SEP and malaria. Six (6) studies were found to have selection bias, and 30 of the 41 studies were cross-sectional, making the claim of mediation only speculative (Additional file 1: Table S4).

## Discussion

This systematic review aimed to identify, summarize and critically appraise the existing evidence regarding the variables that potentially mediate the relationship between SEP and malaria in SSA. Our review shows that evidence of mediating pathways between household SES and malaria is sparse and under-researched.

Of the 41 studies, only two assessed mediators of the SEP and malaria path using formal mediation analyses specifically housing quality, food security, antimalarial use. One study showed that a proportion of the total effect of SEP on malaria was mediated through housing and food security, while another showed minimal mediation by antimalarial use. However, each mediator was only investigated in one study, meaning these findings remain inconclusive. Other studies indicated that ITN use, higher education, better nutrition, housing quality, IRS, and repellent use could, to a great extent, protect against malaria. These, however, did not conduct mediation analyses but included potential mediators as covariates. This review provides valuable insights for directed action/interventions to alleviate poverty-related malaria burdens, improve health outcomes of marginalized populations, and contribute to reducing global malaria incidence and mortality rates by at least 90%, as per the global technical strategy for malaria [61] and in line with the Sustainable Development Goals (SDGs), particularly the goal of ending poverty (SDG 1) and achieving universal health coverage (SDG 3). For instance, based on these findings, interventions that target housing improvements and food security could substantially prevent /mitigate malaria risk. It is important to acknowledge that the measurement of SEP varied across different studies and countries, particularly in terms of the included assets, data reduction techniques, and the decision to categorize or not which significantly impacts the comparability of findings, even when there is consistency in the direction of the association between SEP and malaria. We also acknowledge that SEP may have improved or declined in the study areas however all studies (except one) were published within 10 years since recruitment which makes findings relevant.

### Mediators identified through formal mediation

Our review indicates that housing quality and food security could mediate socioeconomic differences in malaria risk. One cohort study demonstrated that housing quality and food insecurity mediated 24.9% and 18.6% of the effect of SEP on malaria incidence in SSA, respectively [1]. The study also suggested that treatment-seeking behavior could have a mediating role; however, the authors did not perform a robust mediation analysis due to insufficient sample size. The analyses in Tusting’s paper [1] were informed by a conceptual framework that, although commendable, did not operationalize pathways such as access to healthcare and ITNs in their mediation analyses. In another study [46], antimalarial treatment was also found to mediate the association between SEP and malaria. Yet, this study did not provide information on the percentage of total effect mediated and the identifiability assumptions checked, hence requiring a cautious interpretation of the findings [46]. These assumptions are (i) No unmeasured exposure-outcome confounding given covariates, C, (ii) No unmeasured mediator-outcome confounding given C, (iii) No unmeasured exposure-mediator confounding given C, (iv) No effect of exposure that confounds the mediator-outcome relationship [14]. The proportion of the socioeconomic differences in malaria mediated by housing and food security was small (less than 30%), which indicated that other potential mediators could explain part of the effect of SEP. Nevertheless, incremental improvements in housing quality and interventions, such as irrigation and agriculture could promote food security, thereby protecting against malaria.

While previous literature suggests the mediating role of socioeconomic and structural factors in the association between SEP and malaria, research remains limited. Some assumptions for mediation were not always met (including potential reverse causality and identifiability), and many analyses do not consider interactions nor adjust for mediator outcome confounding, which raises concerns about the validity of the mediation effects reported. Future studies need to apply more robust analyses on longitudinal data for which many assumptions may hold.

### Potential mediators (with no formal mediation)

Rather than formal mediation analysis, variables were considered potential mediators if their inclusion in the adjusted models resulted into change (reduction) in the SEP coefficient. While it is generally not recommended to control for mediating variables in the causal relationship because conditioning on them introduces bias [62], the attenuation in coefficients of SEP in a multivariable model implies they could be mediators.

Most studies in this review indicated a protective effect on malaria with higher education [28, 34, 37, 41, 42, 51, 57], IRS [32, 35, 36, 42], better housing [20, 21, 25–27, 29, 41, 43], and use of ITNs after adjusting for SEP. Consistent with previous literature, which shows a consistent association between wealth and ITN use and IRS [63–66], a recent systematic review of the effectiveness of ITNs showed a strong protective effect against malaria [67]. In another review, the addition of IRS on averaged reduced malaria parasite prevalence (*RR* = 0.61, 95% *CI* 0.42 to 0.88) [68] indicates that interventions targeting IRS and ITNs combined may significantly affect malaria morbidity although this effect may not be observed in all contexts. It is important to note that utilization of IRS and ITNs may be affected by their high costs, low coverage and poor quality of IRS in some settings [69].

Educational attainment is also a well-known predictor of malaria risk [6]. Greater wealth encourages higher educational attainment [70], which may increase individuals’ knowledge of prevention and treatment, decision-making, and access to information [58]. This could encourage the uptake of preventive measures and consequently lower malaria risk. In our review, 12 of 18 studies indeed found an association between higher educational attainment and a lower risk of malaria after adjusting for SEP. However, the evidence regarding the proportion of SEP effect mediated through education is limited.

Further, most studies that adjusted for housing quality found that improved housing was protective against malaria with a reduced coefficient of SEP. Higher SEP makes it easier to acquire better housing (shelters with better roofs, shutters, and eaves), which can then reduce exposure to the biting *Anopheles* at night by preventing the entry of mosquito vectors. Evidence for the effect of SEP mediated through housing is still forthcoming, with a single study suggesting that improvements in housing could partly explain the protective effect of SEP [1]. In light of this evidence, improving housing or improving accessibility of building materials to households with low SEP could contribute to reduction of malaria burden. However, there is a need to determine potential mediators and their relative contributions to the association between SEP and malaria to inform the design and implementation of targeted socio-structural interventions against malaria.

## Strengths, limitations and implications for further research

To the best of our knowledge, this is the first systematic review that has attempted to explore the potential mediators on the path between SEP and malaria in SSA. Unlike recent reviews [4, 6], which included education and housing as proxies for SEP, we defined our exposure (SEP) based on household wealth indices (asset-based indices), which is a more reliable measure of household wealth in low-income countries. However, the review’s findings are not without limitations. First, while the review was comprehensive and involved 41 articles and tens of thousands of participants, we identified only three formal mediation analyses of pathways linking SEP and malaria; hence, uncertainties remain around the relative contribution of several potential mediators. Second, we may have missed other studies because we searched only two databases and also did not search grey literature or studies in languages other than English. Nevertheless, we think this is still a specialized area of academic research, and most of the studies that meet the criteria are most likely to be published in international peer-reviewed journals. Thirdly, most studies had methodological limitations, such as the lack of a conceptual framework, sensitivity analyses, ignorability assumptions, and the use of cross-sectional data, which renders claims of causal mediation speculative because temporarity could not be established. Longitudinal designs are better suited to demonstrate temporality, a key aspect in causal inferences and especially important for studies on SEP and malaria due to the bi-directionality of their relationship [30]. With longitudinal data, more suitable methods, such as VanderWeele’s parametric mediational g-formula, can account for time-varying exposures, mediators and confounding affected by previous exposure could be applied [71].

## Conclusions

Our study indicates that a relatively small body of research has tested indirect pathways between SEP and malaria. From reviewed evidence, extant research suggests that housing, food security and recent antimalarial use are likely mediators in the SEP-malaria relationship in SSA. Although other pathways, such as education, IRS and ITN use, nutrition, and health-seeking behaviour, are not fully supported by current evidence, their role cannot be ignored due to their demonstrated protective effect on malaria when modelled with SEP as a covariate. More formal mediation analyses using longitudinal data are needed to overcome methodological limitations, such as cross-sectional data, insufficient confounder adjustment, and limited use of sound conceptual frameworks. This research area holds much potential in informing the design of more effective interventions for malaria control.

## Supplementary Information

**Additional file 1: Table S1.** Highlights of the review. **Table S2.** Detailed search strategy, conducted on May 31, 2022. **Table S3.** Quality assessment tool for quantitative studies. **Table S4.** Assessment of the quality of included studies.

## Data Availability

All data relevant to the study are included in this published article and uploaded as Additional file.

## Abbreviations

DRC: Democratic Republic of Congo
EPHPP: Effective Public Health Practice Project Tool
GEE: Generalized estimating equations
HH: Household
HPD: Highest posterior density
IPTP: Intermittent preventive treatment in pregnancy
IRS: Indoor residual spraying
*IRR*: Incidence rate ratios
ITN: Insecticide treated Nets
*OR*: Odds ratios
RDT: Rapid diagnostic test
PCA: Principal Component Analysis
SEP: Socioeconomic position
SSA: Sub-Saharan Africa
