# Supplementary material for: What are the pathways between poverty and malaria in sub-Saharan Africa? A systematic review of mediation studies
Source: Infect Dis Poverty. 2023 Jun 8;12:58. doi: 10.1186/s40249-023-01110-2 (PMC10249281; doi:10.1186/s40249-023-01110-2)
Supplement: Supplementary file 1 — Additional file 1: Table S1. Highlights of the review. Table S2. Detailed search strategy, conducted on May 31, 2022. Table S3. Quality assessment tool for quantitative studies. Table S4. Assessment of the quality of included studies. [file 40249_2023_1110_MOESM1_ESM.docx]

## **Table S1:** Highlights of the review.

| What is already known about this topic | - Recent reviews have consistently found associations between socioeconomic position (SEP) and malaria using different definitions of SEP. - Thus far, no review had explored putative mediators between SEP and malaria risk. |
| --- | --- |
| What this study adds | - The review is the first to explore mediators between SEP and Malaria. - It expands our understanding of the mechanisms through which SEP creates disparities in malaria risk by building on an extensive systematic literature research and examining available evidence on the relative contribution of mediators on the different pathways linking SEP and malaria. - Findings suggest that food security and housing investments could be feasible targets for structural interventions against malaria. |
| How this study might affect research, practice or policy | - Our review identifies limited research on the mediating role of variables on the pathway from SEP to malaria. It highlights a need for further high-quality longitudinal empirical research using mediation analysis. |

## **Table S2:** Detailed search strategy, conducted on May 31, 2022.

| MEDLINE (Pubmed):  "(malaria OR plasmodium OR fever) AND (socioeconomic status OR socio-economic status OR socioeconomic position OR socio-economic position OR income OR wealth OR poverty OR equity OR house* OR employment* OR occupation* OR education*) AND (mediator OR mediation OR Path*) AND (sub saharan africa OR sub-saharan africa OR ssa OR angola OR benin OR botswana OR burkina faso OR burundi OR cameroon OR cabo verde OR cape verde OR central african republic OR chad OR comoros OR democratic republic of congo OR congo OR cote d'ivoire OR djibouti OR equatorial guinea OR eritrea OR eswatini OR swaziland OR ethiopia OR gabon OR the gambia OR ghana OR guinea OR guinea-bissau OR kenya OR lesotho OR liberia OR madagascar OR malawi OR mali OR mauritania OR mauritius OR mayotte OR mozambique OR namibia OR niger OR nigeria OR réunion OR rwanda OR sao tome and principe OR senegal OR seychelles OR sierra leone OR somalia OR south africa OR south sudan OR sudan OR swaziland OR tanzania OR togo OR uganda OR zambia OR zimbabwe)” |
| --- |
| Web of Science:  In Web of Science following databases were included: Web of Science, Biosis Citation Index (BCI), Current Conten Connect (CCC), Data Citation Index (DRCI), Korean Journal Databank (KJD), Medline (Pubmed), Russian Science Citation Index (RSCI), and Scielo Citation Index.  “TS=((malaria OR  plasmodium  OR  fever)  AND  (socioeconomic status OR socioeconomic position OR income OR wealth OR poverty OR equity OR house* OR employment* OR occupation* OR education*)  AND  (mediator OR mediation OR Path*) )  AND  (CU= (sub Saharan africa OR sub-saharan africa OR ssa OR angola OR benin OR botswana OR burkina faso OR burundi OR cameroon OR cabo verde OR cape verde OR central african republic OR chad OR comoros OR democratic republic of congo OR congo OR cote d'ivoire OR djibouti OR equatorial guinea OR eritrea OR eswatini OR swaziland OR ethiopia OR gabon OR the gambia OR ghana OR guinea OR guinea-bissau OR kenya OR lesotho OR liberia OR madagascar OR malawi OR mali OR mauritania OR mauritius OR mayotte OR mozambique OR namibia OR niger OR nigeria OR réunion OR rwanda OR sao tome and principe OR senegal OR seychelles OR sierra leone OR somalia OR south africa OR south sudan OR sudan OR swaziland OR tanzania OR togo OR uganda OR zambia OR zimbabwe) )  Databases= WOS, BCI, CCC, DRCI, KJD, MEDLINE, RSCI, SCIELO Timespan=2000-2022 Search language=English” |

| **Table S3**. Quality assessment tool for quantitative studies- (Adapted) |
| --- |
| SELECTION BIAS (Q1) Are the individuals selected to participate in the study likely to be representative of the target population?   - 1. Very likely   2. Somewhat likely   3. Not likely   4. Can’t tell   (Q2) What percentage of selected individuals agreed to participate?   1. 80 - 100% agreement 2. 60 – 79% agreement 3. less than 60% agreement 4. Not applicable 5. Can’t tell  \| **RATE THIS SECTION** \| **STRONG** \| **MODERATE** \| **WEAK** \| \| --- \| --- \| --- \| --- \| \| See dictionary \| 1 \| 2 \| 3 \| |
| STUDY DESIGN Indicate the study design   1. Randomized controlled trial 2. Controlled clinical trial 3. Cohort analytic (two group pre + post) 4. Cohort (one group pre + post (before and after)) 5. Case-control 6. Cross sectional study 7. Can’t tell   Was the study described as randomized? If NO, go to Component C.  No Yes  If Yes, was the method of randomization described? (See dictionary)  No Yes  If Yes, was the method appropriate? (See dictionary)  No Yes   \| **RATE THIS SECTION** \| **STRONG** \| **MODERATE** \| **WEAK** \| \| --- \| --- \| --- \| --- \| \| **See dictionary** \| 1 \| 2 \| 3 \| |
| CONTROL OF CONFOUNDING (Q1) Did the authors attempt to control for cofounders ?   1. Yes 2. No 3. Can’t tell   The following are examples of confounders:   1. Education 2. Income 3. Sex 4. Age 5. ITN use OR IRS use 6. Antimalaria treatment 7. Health seeking behavior   (Q2) If yes, indicate the percentage of relevant confounders that were controlled (either in the design (e.g. stratification, matching) or analysis)?  1 80 – 100% (most)  2 60 – 79% (some)   1. Less than 60% (few or none) 2. Can’t Tell  \| **RATE THIS SECTION** \| **STRONG** \| **MODERATE** \| **WEAK** \| \| --- \| --- \| --- \| --- \| \| **See dictionary** \| 1 \| 2 \| 3 \| |
| Exposure measurement (Q1) Was the measurement of the exposure done in a way to minimize bias? (Check consistency and validity of method)   1. Yes 2. No, 3. No /Can’t tell  \| **RATE THIS SECTION** \| **STRONG** \| **MODERATE** \| **WEAK** \| \| --- \| --- \| --- \| --- \| \| **If Q1** \| 1 \| 2 \| 3 \| |
| Outcome measurement (Q1) Was the measurement of the outcome done in a way to minimize bias? (Check consistency and validity of method)   1. Yes (objective /blinded) 2. No (self-report, not measured using reference standard) 3. Can’t tell or Not mentioned   For cohort, check length of follow up-long enough?   \| **RATE THIS SECTION** \| **STRONG** \| **MODERATE** \| **WEAK** \| \| --- \| --- \| --- \| --- \| \| **If** \| 1 \| 2 \| 3 \| |
| WITHDRAWALS AND DROP-OUTS (prospective designs) (Q1) Were withdrawals and drop-outs reported in terms of numbers and/or reasons per group?   1. Yes 2. No 3. Can’t tell 4. Not Applicable (i.e. one time surveys or interviews)   (Q2) Indicate the percentage of participants completing the study. (If the percentage differs by groups, record the lowest).  1 80 -100%  2 60 - 79%   1. less than 60% 2. Can’t tell 3. Not Applicable (i.e. Retrospective case-control)  \| **RATE THIS SECTION** \| **STRONG** \| **MODERATE** \| **WEAK** \|  \| \| --- \| --- \| --- \| --- \| --- \| \| **See dictionary** \| 1 \| 2 \| 3 \| Not Applicable \| |
| GLOBAL RATING  See dictionary on how to rate this section.   \| **A** \| **SELECTION BIAS** \| **STRONG** \| **MODERATE** \| **WEAK** \|  \| \| --- \| --- \| --- \| --- \| --- \| --- \| \|  \|  \| 1 \| 2 \| 3 \|  \| \| **B** \| **STUDY DESIGN** \| **STRONG** \| **MODERATE** \| **WEAK** \|  \| \|  \|  \| 1 \| 2 \| 3 \|  \| \| **C** \| **CONTROL OF CONFOUNDING** \| **STRONG** \| **MODERATE** \| **WEAK** \|  \| \|  \|  \| 1 \| 2 \| 3 \|  \| \| **D** \| **EXPOSURE MEASUREMENT** \| **STRONG** \| **MODERATE** \| **WEAK** \|  \| \|  \|  \| 1 \| 2 \| 3 \|  \| \| **E** \| **OUTCOME MEASUREMENT** \| **STRONG** \| **MODERATE** \| **WEAK** \|  \| \|  \|  \| 1 \| 2 \| 3 \|  \| \| **F** \| **WITHDRAWALS AND DROPOUTS** \| **STRONG** \| **MODERATE** \| **WEAK** \|  \| \|  \|  \| 1 \| 2 \| 3 \| Not Applicable \|  GLOBAL RATING FOR THIS PAPER (circle one):  \| 1 \| STRONG \| (no WEAK ratings) \| \| --- \| --- \| --- \| \| 2 \| MODERATE \| (One WEAK rating) \| \| 3 \| WEAK \| (two or more WEAK ratings) \|   With both reviewers discussing the ratings:  Is there a discrepancy between the two reviewers with respect to the component (A-F) ratings?  No Yes  If yes, indicate the reason for the discrepancy   1. Oversight 2. Differences in interpretation of criteria 3. Differences in interpretation of study  \| **Final decision of both reviewers (circle one):** \| **1** \| **STRONG** \| \| --- \| --- \| --- \| \|  \| **2** \| **MODERATE** \| \|  \| **3** \| **WEAK** \| |

**Table S4**. Assessment of the quality of included studies (based on adapted EPHPP tool)

| Study  no. | Author (Year) | Selection  bias | Study  design | Control of confounding | Exposure measurement | Outcome measurement | Withdrawals and drop-outs | Final rating |
| --- | --- | --- | --- | --- | --- | --- | --- | --- |
| 1 | Chirombo et al. 2014 | 2 | 3 | 3 | 2 | 2 | NA | Weak |
| 2 | De Granville et al. 2019 | 1 | 3 | 2 | 1 | 1 | NA | Moderate |
| 3 | Florey et al. 2012 | 3 | 3 | 3 | 1 | 1 | N/A | Weak |
| 4 | Gari et al. 2016 | 1 | 2 | 2 | 2 | 1 | 1 | Strong |
| 5 | Haji et al. 2016 | 2 | 3 | 1 | 2 | 1 | NA | Moderate |
| 6 | Kabaghe et al. 2017 | 1 | 3 | 3 | 2 | 2 | NA | Weak |
| 7 | Kahabuka et al. 2012 | 2 | 3 | 3 | 2 | 2 | NA | Weak |
| 8 | Liu et al. 2014 | 1 | 1 | 2 | 1 | 1 | 1 | Strong |
| 9 | Ma et al. 2017 | 1 | 3 | 2 | 2 | 1 | NA | Moderate |
| 10 | Mathanga et al 2015 | 1 | 3 | 2 | 2 | 1 | NA | Moderate |
| 11 | Sakwe et al. 2019 | 2 | 3 | 1 | 2 | 1 | NA | Moderate |
| 12 | Siri et al. 2010 | 2 | 2 | 2 | 2 | 1 | NA | Strong |
| 13 | Skarbinski et al. 2011 | 2 | 3 | 3 | 1 | 1 | NA | weak |
| 14 | Skarbinski et al. 2012 | 1 | 3 | 3 | 1 | 1 | NA | Weak |
| 15 | Snyman et al. 2015 | 3 | 2 | 2 | 1 | 1 | 2 | moderate |
| 16 | Somi et al.2007 | 2 | 3 | 1 | 2 | 1 | NA | Moderate |
| 17 | Somi et al. 2008 | 1 | 3 | 2 | 1 | 1 | NA | Moderate |
| 18 | Ssempiira et al. 2017 | 1 | 3 | 1 | 1 | 2 | NA | Moderate |
| 19 | Temu et al. 2012 | 3 | 3 | 2 | 2 | 2 | NA | Weak |
| 20 | Tusting et al. 2016 | 2 | 2 | 2 | 1 | 1 | 2 | Strong |
| 21 | Wanzirah et al. 2015 | 2 | 2 | 2 | 2 | 1 | 2 | Strong |
| 22 | West et al. 2013 | 1 | 3 | 2 | 1 | 2 | NA | moderate |
| 23 | William et al. 2016 | 1 | 3 | 3 | 2 | 1 | NA | Weak |
| 24 | Zgambo et al. 2017 | 1 | 3 | 2 | 2 | 1 | NA | Moderate |
| 25 | Zoungrana et al. 2014 | 2 | 3 | 1 | 2 | 1 | NA | Moderate |
| 26 | Loha et al. 2012 | 1 | 2 | 2 | 2 | 2 | 1 | strong |
| 27 | Graves et al. 2009 | 1 | 3 | 2 | 2 | 2 | NA | Moderate |
| 28 | Coleman et al. 2010 | 1 | 2 | 3 | 2 | 1 | NA | Moderate |
| 29 | Mmbando et al. 2011 | 1 | 3 | 2 | 2 | 1 | NA | moderate |
| 30 | Clark et al. 2008 | 1 | 2 | 2 | 2 | 1 | 2 | Strong |
| 31 | Siri 2014 | 1 | 3 | 2 | 2 | 1 | NA | Moderate |
| 32 | Custodio et al. 2009 | 1 | 3 | 2 | 2 | 1 | NA | Moderate |
| 33 | De Beaudrap et al. 2011 | 1 | 3 | 2 | 2 | 1 | NA | moderate |
| 34 | Asante et al. 2013 | 1 | 2 | 2 | 2 | 1 | 2 | Strong |
| 35 | Sonko et al. 2014 | 1 | 3 | 2 | 2 | 1 | NA | Moderate |
| 36 | Vincenz et al. 2022 | 3 | 2 | 3 | 2 | 1 | 3 | Weak |
| 37 | Mann D et al. 2021 | 1 | 3 | 2 | 1 | 1 | NA | Moderate |
| 38 | Emina JB et al. 2021 | 2 | 3 | 2 | 1 | 1 | NA | Moderate |
| 39 | Mwaiswelo et al. 2021 | 3 | 3 | 2 | 1 | 2 | NA | Weak |
| 40 | Mangani et al. 2022 | 1 | 3 | 1 | 1 | 2 | NA | Moderate |
| 41 | Ejigu 2020 | 3 | 3 | 2 | 1 | 2 | NA | Weak |
